# An Inexact Optimization Model for Crop Area Under Multiple Uncertainties

**DOI:** 10.3390/ijerph16142610

**Published:** 2019-07-22

**Authors:** Chongfeng Ren, Hongbo Zhang

**Affiliations:** 1 Key Laboratory of Subsurface Hydrology and Ecological Effect in Arid Region, Ministry of Education, Xi’an 710054, China; 2 School of Environmental Science and Engineering, Chang’an Univeristy, Xi’an 710054, China

**Keywords:** crop area optimization, multiple uncertainties, chance constrained programming, type-2 fuzzy interval programming, water-saving scenarios

## Abstract

This paper developed a type-2 fuzzy interval chance constrained programming model for optimizing a crop area, which integrated chance constrained programming and type-2 fuzzy interval programming. The developed model was then applied to a case study in Wuwei City, Gansu Province, China, and the maximization of economic benefit was selected as the planning objective. Furthermore, different water-saving irrigation modes were considered as the development mode. A series of optimal irrigation water and planting structure schemes were obtained under different violation probabilities in each water-saving scenario. The obtained results could be helpful to make decisions on the planting structure and the optimal use of irrigation water and land resources under multiple uncertainties.

## 1. Introduction

Water resources play an irreplaceable role in life on earth. Water resources are not only basic natural resources but also strategic economic resources and ecological control factors [1,2]. However, water resource shortages are becoming increasingly serious, while the demand for water resources is increasing with the development of economies and societies. In addition, human health has been threatened by water resource shortages. For example, there are approximately 700 million people that are suffering from water scarcity. Furthermore, 1.8 billion people will be facing severe water scarcity by 2025 according to the United Nations [3].

Although water resource shortages are worsening, there is also a great waste of water resources, especially in agriculture [4,5]. For example, the effective utilization rate of agricultural irrigation water is only approximately 0.5 in China, which is one of the thirteen most water-deficient countries. This also indicates that agricultural water management, which can improve the efficiency of agricultural water usage, can play an important role in agricultural water consumption. Moreover, the crop area allocation, which has a close correlation with the planting structure and agricultural water consumption, can be used as an effective indicator when optimizing agricultural water resources [6,7]. Therefore, a potential scheme for optimizing the allocation of agricultural water can be determined by optimizing the crop area allocation.

In the last few decades, there have been many studies on optimizing crop areas [8,9,10,11,12,13,14]. For example, Nguyen et al. 2016 developed an improved ant colony optimization formulation for the allocation of crops and water to different irrigation areas, which facilitate the identification of better solutions at all stages of the search and reduce the computational time by using visibility factors and dynamic decision variable options [15]. To deal with the major challenge of meeting the growing food demand with minimum environmental impact, a novel systematic method was presented for agricultural planning that optimally allocates the rainfed and irrigated cropping areas, thereby enhancing food availability and reducing the environmental impact of agriculture [16]. To determine the optimum crop patterns, a network flow programming model, which was based on a simulation model and the shuffled frog leaping optimizing algorithm in the form of a simulation-optimization approach, was developed [17].

Although the crop area or crop planting structure has been optimized by establishing a series of crop optimization models, many parameters that are ignored in the above optimization models have uncertainty and cannot present precise actual values [2,18,19]. In recent years, studies on the optimal allocation of crop areas considered these uncertainties. For example, a fuzzy dynamic programming model was developed for crop planning and the conjunctive use of surface water and groundwater by Hamid and Mohammad [20]. To deal with the uncertain parameters involved in the optimal allocation of crop areas, an optimal model was established, which introduced the interval and functional interval uncertainty methods in the crop water production function [21]. Xie et al. 2013 developed an inexact two-stage stochastic programming model for planning agricultural irrigation, which took four planning districts, four water users, and five water sources into consideration [22]. To deal with the significant challenge, the inherent uncertainties and their potential interactions, Wang and Huang. 2014 proposed a methodology that incorporated optimization techniques and statistical experimental designs within a general framework to address the issues of uncertainty and risk as well as their correlations in a systematic manner [23]. A process-based regional economic optimization model that was developed in response to water scarcity has been a crucial development for sustainable irrigated agriculture in arid regions; this was a two-level optimization model with combined use as an agro-hydrological model [24].

Although many optimization methods, which mainly focused on a single uncertainty, were developed by the above studies, they cannot deal with the complex uncertainty parameters involving multiple uncertainties [25,26]. For example, in reality, uncertainties may exist in the membership function of fuzzy mathematical programming (FMP). As a result, a fuzzy set may be present, resulting in a type-2 fuzzy set (T2FS) [27,28,29,30,31,32,33]. In addition, when facing T2FS problems, uncertainties may exist in the objective function or left-hand constraints. Therefore, the type-2 fuzzy interval programming (T2FIP) method was introduced to deal with the complex uncertain parameters in the optimal allocation of crop areas.

Furthermore, during the process of optimizing the crop area, a series of parameters have random characteristics and can be expressed as probability distributions, such as the available surface water resources, rainfall and water resource storages [34,35]. The chance-constrained programming (CCP) method has the ability to deal with random parameters in the right-hand-side constraints [35,36,37]. Therefore, the CCP model was introduced to deal with the random parameters in the optimal allocation of crop areas.

Therefore, in order to deal with the complex uncertain parameters and multiple uncertainties in the process of optimizing crop areas, this paper developed an improved type-2 fuzzy interval chance constrained programming (T2FICCP) by integrating T2FIP and CCP. In addition, the developed model was also applied to Wuwei City, Gansu Province, China. Furthermore, both the conventional irrigation mode and water-saving mode exist in the planting industry of Wuwei City. Thus, different water-saving scenarios were constructed by setting different water-saving levels in the established model. Compared with the previous studies, the proposed model has the following advantages: (a) it can deal with the complex uncertain parameters which have the characteristics of T2FS and interval numbers simultaneously; (b) it can handle the multiple uncertainties expressed as T2FS, stochastics and intervals, (c) it can take food security, groundwater exploitation and the available surface water into account, (d) a series of optimal schemes under multiple uncertainties of each water-saving scenario can be provided to decision makers. Generally, a desired optimal scheme, that allocates the crop area and agricultural water under multiple uncertainties and multiple water-saving scenarios, could be identified for the decision makers by the developed model.

## 2. Model Formulation

### 2.1. Type-2 Fuzzy Interval Programming

The type-2 fuzzy interval programming (T2FIP) models are formulated as follows:

(1) $$Max{\tilde{f}}^{\pm }=A^{\pm }X^{\pm }$$

Subject to

(2) $$C_{1}^{\pm }X^{\pm }≾{\tilde{D}}_{1}^{\pm }$$

(3) $$C_{2}^{\pm }X^{\pm }≾{\tilde{D}}_{2}^{\pm }$$

(4) $$X^{\pm }\geq 0$$

where, $C_{1}^{\pm }\in {\left\{R^{\pm }\right\}}^{s\times n}$, $C_{2}^{\pm }\in {\left\{R^{\pm }\right\}}^{\left(m-s\right)\times n}$, ${\tilde{D}}_{1}^{\pm }\in {\left\{R^{\pm }\right\}}^{m\times 1}$, ${\tilde{D}}_{2}^{\pm }\in {\left\{R^{\pm }\right\}}^{\left(m-s\right)\times 1}$, $A^{\pm }\in {\left\{R^{\pm }\right\}}^{n\times 1}$. $R^{\pm }\;$ is the set of interval numbers; $R^{\pm }\;$ is a T2FS set characterized by its two primary membership functions; *m* is a row vector with m elements; *s* is a row vector with s elements; *n* is a column vector with n elements. In addition, ≾ denotes a fuzzy partial order. The established model in Equation (1), can be divided into two submodels with type-2 fuzzy parameters, when the upper and lower bound of two T2FSs in the same interval do not intersect and there is independence between the upper bound and lower bound of the T2FS. In addition, the two fuzzy optimization submodels can be obtained by introducing the penalty coefficient which has the ability to transform the T2FS to conventional fuzzy sets. Finally, the two transformed submodels can become two linear models which can be easily solved when system satisfaction was introduced. When the objective function is a maximization, the upper bound submodel (${\tilde{f}}^{\pm }$) should be solved first; then the lower bound submodel (${\tilde{f}}^{-}$) can be solved based on the solution of upper bound submodel [27]. The detailed solution of the upper bound submodel $({\tilde{f}}^{+}$) is as follows [26,30]:

(5) $$Max{\tilde{f}}^{\pm }=\overset{k}{\underset{j=1}{\sum }}a_{j}^{+}\cdot x_{j}^{+}+\overset{n}{\underset{j=k+1}{\sum }}a_{j}^{+}\cdot x_{j}^{-}$$

Subject to

(6) $$\overset{k}{\underset{j=1}{\sum }}{|c_{rj}|}^{-}\mathrm{Sign}\left(c_{rj}^{-}\right)x_{j}^{+}+\overset{n}{\underset{j=k+1}{\sum }}{|c_{rj}|}^{+}\mathrm{Sign}\left(c_{rj}^{+}\right)x_{j}^{-}≾{\tilde{d}}_{r}^{+},\;\;\;r=1,2\ldots ,s$$

(7) $$\overset{k}{\underset{j=1}{\sum }}{|c_{tj}|}^{-}\mathrm{Sign}\left(c_{tj}^{-}\right)x_{j}^{+}+\overset{n}{\underset{j=k+1}{\sum }}{|c_{tj}|}^{+}\mathrm{Sign}\left(c_{tj}^{+}\right)x_{j}^{-}≾{\tilde{d}}_{t}^{+},\;\;\;t=s+1,\;s+2,\ldots ,m$$

(8) $$x_{j}^{+}\geq 0,\;\;j=1,2,\ldots ,k$$

(9) $$x_{j}^{-}\geq 0,\;\;j=k+1,\;k+2,\ldots ,n$$

where, ${|c_{rj}|}^{+}\;$ and ${|c_{rj}|}^{-}$ represent the upper and lower bound absolute values of $c_{rj}^{\pm }$, respectively. In addition, $\mathrm{Sign}\left(c_{tj}^{-}\right)$ represents the sign of $c_{tj}^{-}$ which can be positive or negative. $x_{j}^{+}\left(j=1,2,\ldots ,k\right)$ and $x_{j}^{-}\left(j=k+1,\;k+2,\ldots ,n\right)$ represent the positive and negative coefficients of the decision variables in the objective function, respectively. Furthermore, $b_{r1u}$ and $b_{r2u}$, the intermediate variables, are introduced in the elastic constraints, $q_{r1u}$ and $q_{r2u}$, the penalty coefficients, are embedded in the objective function, which follows the T2FS optimization method [26,30]. Therefore, Equations (5) and (6) can be transformed into two submodels which are follows:

(10) $$Maxf_{l}^{+}=\overset{k}{\underset{j=1}{\sum }}a_{j}^{+}\cdot x_{j}^{+}+\overset{n}{\underset{j=k+1}{\sum }}a_{j}^{+}\cdot x_{j}^{-}$$

Subject to:

(11) $$\overset{k}{\underset{j=1}{\sum }}{|c_{rj}|}^{-}\mathrm{Sign}\left(c_{rj}^{-}\right)x_{j}^{+}+\overset{n}{\underset{j=k+1}{\sum }}{|c_{rj}|}^{+}\mathrm{Sign}\left(c_{rj}^{+}\right)x_{j}^{-}≾d_{r3}^{+},\;\;\;r=1,2\ldots ,s$$

(12) $$\overset{k}{\underset{j=1}{\sum }}{|c_{tj}|}^{-}\mathrm{Sign}\left(c_{tj}^{-}\right)x_{j}^{+}+\overset{n}{\underset{j=k+1}{\sum }}{|c_{tj}|}^{+}\mathrm{Sign}\left(c_{tj}^{+}\right)x_{j}^{-}≾d_{t}^{+},\;\;\;t=s+1,\;s+2,\ldots ,m$$

(13) $$x_{j}^{+}\geq 0,\;\;j=1,2,\ldots ,k$$

(14) $$x_{j}^{-}\geq 0,\;\;j=k+1,\;k+2,\ldots ,n$$

And:

(15) $$Maxf_{u}^{+}=\overset{k}{\underset{j=1}{\sum }}a_{j}^{+}\cdot x_{j}^{+}+\overset{n}{\underset{j=k+1}{\sum }}a_{j}^{+}\cdot x_{j}^{-}-\overset{s}{\underset{r=1}{\sum }}b_{r1u}\cdot q_{r1u}-\overset{s}{\underset{r=1}{\sum }}b_{r2u}\cdot q_{r2u}$$

Subject to:

(16) $$\overset{k}{\underset{j=1}{\sum }}{\left|c_{rj}\right|}^{-}\mathrm{Sign}\left(c_{rj}^{-}\right)x_{j}^{+}+\overset{n}{\underset{j=k+1}{\sum }}{\left|c_{rj}\right|}^{+}\mathrm{Sign}\left(c_{rj}^{+}\right)x_{j}^{-}-b_{r1u}\geq d_{r1}^{+},\;\;\;r=1,2\ldots ,s$$

(17) $$\overset{k}{\underset{j=1}{\sum }}{\left|c_{rj}\right|}^{-}\mathrm{Sign}\left(c_{rj}^{-}\right)x_{j}^{+}+\overset{n}{\underset{j=k+1}{\sum }}{\left|c_{rj}\right|}^{+}\mathrm{Sign}\left(c_{rj}^{+}\right)x_{j}^{-}-b_{r2u}\leq d_{r4}^{+},\;\;\;r=s+1,s+2\ldots ,s$$

(18) $$\overset{k}{\underset{j=1}{\sum }}{\left|c_{tj}\right|}^{-}\mathrm{Sign}\left(c_{tj}^{-}\right)x_{j}^{+}+\overset{n}{\underset{j=k+1}{\sum }}{\left|c_{tj}\right|}^{+}\mathrm{Sign}\left(c_{tj}^{+}\right)x_{j}^{-}\leq d_{t}^{+},\;\;\;t=s+1,s+2\ldots ,m$$

(19) $$0\leq b_{r1u}\leq d_{r2}^{+}-d_{r1}^{+},\;r=1,2,\ldots ,s$$

(20) $$0\leq b_{r2u}\leq d_{r5}^{+}-d_{r4}^{+},\;r=1,2,\ldots ,s$$

(21) $$x_{j}^{+}\geq 0,\;\;j=1,2,\ldots ,k$$

(22) $$x_{j}^{-}\geq 0,\;\;j=k+1,k+2,\ldots ,n$$

where, $f_{l}^{+}$ and $f_{u}^{+}$ represent the upper and lower bound values of the upper submodel, respectively. In addition, $f_{l}^{+}$, $f_{u}^{+}$, $d\prime_{r1}^{+}=d_{r1}^{+}+b_{r1u}$ and $d\prime_{r4}^{+}=d_{r4}^{+}+b_{r2u}$ can be introduced into the model (5–9) based on the established fuzzy objective membership function. Therefore, the upper bound submodel can be transformed as follows:

(23) $$Max\;\lambda_{1}$$

Subject to:

(24) $$\overset{k}{\underset{j=1}{\sum }}a_{j}^{+}\cdot x_{j}^{+}+\overset{n}{\underset{j=k+1}{\sum }}a_{j}^{+}\cdot x_{j}^{-}-\lambda_{1}(f_{u}^{+}-f_{l}^{+})\geq f_{l}^{+}$$

(25) $$\overset{k}{\underset{j=1}{\sum }}{\left|c_{rj}\right|}^{-}\mathrm{Sign}\left(c_{rj}^{-}\right)x_{j}^{+}+\overset{n}{\underset{j=k+1}{\sum }}{\left|c_{rj}\right|}^{+}\mathrm{Sign}\left(c_{rj}^{+}\right)x_{j}^{-}-\lambda_{1}\left(d_{r3}^{+}-d\prime_{r1}^{+}\right)\geq d\prime_{r1}^{+},\;\;\;r=1,2\ldots ,s$$

(26) $$\overset{k}{\underset{j=1}{\sum }}{\left|c_{rj}\right|}^{-}\mathrm{Sign}\left(c_{rj}^{-}\right)x_{j}^{+}+\overset{n}{\underset{j=k+1}{\sum }}{\left|c_{rj}\right|}^{+}\mathrm{Sign}\left(c_{rj}^{+}\right)x_{j}^{-}-\lambda_{1}\left(d_{r3}^{+}-d\prime_{r4}^{+}\right)\geq d\prime_{r4}^{+},\;\;\;r=1,2\ldots ,s$$

(27) $$\overset{k}{\underset{j=1}{\sum }}{\left|c_{tj}\right|}^{-}\mathrm{Sign}\left(c_{tj}^{-}\right)x_{j}^{+}+\overset{n}{\underset{j=k+1}{\sum }}{\left|c_{tj}\right|}^{+}\mathrm{Sign}\left(c_{tj}^{+}\right)x_{j}^{-}\leq d_{t}^{+},\;\;\;t=s+1,s+2\ldots ,m$$

(28) $$0\leq \lambda_{1}\leq 1$$

(29) $$x_{j}^{+}\geq 0,\;\;j=1,2,\ldots ,k$$

(30) $$x_{j}^{-}\geq 0,\;\;j=k+1,k+2,\ldots ,n$$

where, $\lambda_{1}$ is the control variable. In addition, the solution, $x_{jopt}^{+}\left(j=1,2,\ldots ,k\right)$, $x_{jopt}^{+}\left(j=k+1,\;k+2,\ldots ,n\right)$ and $f_{opt}^{+}$, can be obtained by solving the above model (23–30).

Furthermore, based on the solution process of the upper bound submodel, the lower bound submodel can be transformed as follows:

(31) $$Max\;\lambda_{2}$$

Subject to:

(32) $$\overset{k}{\underset{j=1}{\sum }}a_{j}^{-}\cdot x_{j}^{-}+\overset{n}{\underset{j=k+1}{\sum }}a_{j}^{-}\cdot x_{j}^{+}-\lambda_{2}(f_{u}^{-}-f_{l}^{-})\geq f_{l}^{-}$$

(33) $$\overset{k}{\underset{j=1}{\sum }}{\left|c_{rj}\right|}^{+}\mathrm{Sign}\left(c_{rj}^{+}\right)x_{j}^{-}+\overset{n}{\underset{j=k+1}{\sum }}{\left|c_{rj}\right|}^{-}\mathrm{Sign}\left(c_{rj}^{-}\right)x_{j}^{+}-\lambda_{2}\left(d_{r3}^{-}-d\prime_{r1}^{-}\right)\geq d\prime_{r1}^{-},\;\;\;r=1,2\ldots ,s$$

(34) $$\overset{k}{\underset{j=1}{\sum }}{\left|c_{rj}\right|}^{+}\mathrm{Sign}\left(c_{rj}^{+}\right)x_{j}^{-}+\overset{n}{\underset{j=k+1}{\sum }}{\left|c_{rj}\right|}^{-}\mathrm{Sign}\left(c_{rj}^{-}\right)x_{j}^{+}-\lambda_{2}\left(d_{r3}^{-}-d\prime_{r4}^{-}\right)\geq d\prime_{r4}^{-},\;\;\;r=1,2\ldots ,s$$

(35) $$\overset{k}{\underset{j=1}{\sum }}{\left|c_{tj}\right|}^{+}\mathrm{Sign}\left(c_{tj}^{+}\right)x_{j}^{-}+\overset{n}{\underset{j=k+1}{\sum }}{\left|c_{tj}\right|}^{-}\mathrm{Sign}\left(c_{tj}^{-}\right)x_{j}^{+}\leq d_{t}^{-},\;\;\;t=s+1,s+2\ldots ,m$$

(36) $$0\leq \lambda_{2}\leq 1$$

(37) $$x_{j}^{-}\geq 0,\;\;j=1,2,\ldots ,k$$

(38) $$x_{j}^{+}\geq 0,\;\;j=k+1,k+2,\ldots ,n$$

(39) $$x_{j}^{-}\geq x_{jopt}^{+},\;\;j=1,2,\ldots ,k$$

(40) $$x_{j}^{+}\geq x_{jopt}^{-},\;\;j=k+1,k+2,\ldots ,n$$

The solution, $x_{jopt}^{-}\left(j=1,2,\ldots ,k\right)$, $x_{jopt}^{-}\left(j=k+1,k+2,\ldots ,n\right)$ and $f_{opt}^{-}$, can be obtained by solving the model (6). Therefore, based on the solution of the two submodels, the optimized solution, $x_{jopt}^{+}=\left[x_{jopt}^{-},\;x_{jopt}^{+}\right]$ and objective value, $f_{opt}^{\pm }=\left[f_{opt}^{-},\;f_{opt}^{+}\right]$, of the model (1–4) with T2FS can be obtained.

### 2.2. The Chance Constrained Programming Model

A series of parameters have random characteristics and can be expressed as probability distributions. The chance constrained programming (CCP) model has a great advantage in dealing with cases in which the parameters on the right hand have random characteristics and can be represented by probability distributions [2]. The typical CCP model is as follows:

(41) $$\mathrm{Min}f\left(x\right)$$

(42) $$\mathrm{Pr}\left[A_{i}\left(x\right)\leq b_{i}\left(x\right)\right]\geq 1-P_{i},\;\;i=1,2,\ldots ,m$$

(43) x≥0

where $A_{i}\left(x\right)\in A\left(x\right)$, $b_{i}\left(x\right)\in B\left(x\right)$, x∈X; $A\left(x\right)$ and $B\left(x\right)$ are random in the probability space X; $P_{i}\left(P_{i}\in \left[0,1\right]\right)$ is the probability level of the stochastic event; and m is the number of constraint events.

When solving the above problems by a CCP model, it could be very difficult to obtain the solution because the constraints (Equation (42)) are always nonlinear and there is uncertainty on both sides of the model [38]. However, in practice, the constraint cannot be totally random in a CCP model [39]. Therefore, the constraint (Equation (42)) can be transformed into a linear programming model which has a feasible constraint set when the parameters are deterministic on the left-hand side while the parameters are variable on the right-hand side [2]. This model can be defined as follows:

(44) $$A_{i}\leq b_{i}{\left(x\right)}^{\left(P_{i}\right)},\;\;i=1,2,\ldots ,m$$

where, $b_{i}{\left(x\right)}^{\left(P_{i}\right)}=F_{i}^{-1}\left(P_{i}\right)$. In addition, $b_{i}\left[i.e.,F_{i}\left(b_{i}\right)\right]$ represents the cumulative distribution function; and $P_{i}$ is the probability of violating the constraint.

### 2.3. Type-2 Fuzzy Interval Chance Constrained Programming

Optimizing the allocation of a crop area may involve multiple uncertainties and complex uncertain parameters. As a result, the process of optimizing the crop area could be very complex. Therefore, in order to deal with the above problems, this paper developed an improved type-2 fuzzy interval chance constrained programming (T2FICCP) model by integrating T2FP, IP and CCP, which is formulated as follows:

(45) $$Max{\tilde{f}}^{\pm }=A^{\pm }X^{\pm }$$

Subject to:

(46) $$C_{1}^{\pm }X^{\pm }≾{\tilde{D}}_{1}^{\pm }$$

(47) $$C_{2}^{\pm }X^{\pm }\leq D_{2}^{\pm }$$

(48) $$\mathrm{Pr}\left[C_{i}\left(X\right)\leq d_{i}\left(X\right)\right]\geq 1-P_{i},\;\;i=1,2,\ldots ,m$$

(49) $$X^{\pm }\geq 0$$

The solution process of the established T2FICCP model is that the random parameters should be converted based on the CCP model, first. Therefore, the constraint (48) of the established model is converted into Equation (53), which is described as follows:

(50) $$Max{\tilde{f}}^{\pm }=A^{\pm }X^{\pm }$$

Subject to:

(51) $$C_{1}^{\pm }X^{\pm }≾{\tilde{D}}_{1}^{\pm }$$

(52) $$C_{2}^{\pm }X^{\pm }\leq D_{2}^{\pm }$$

(53) $$C_{i}\leq d_{i}{\left(X\right)}^{\left(P_{i}\right)},\;\;i=1,2,\ldots ,m$$

(54) $$X^{\pm }\geq 0$$

Furthermore, the transformed model (50–54) with T2FS can be solved based on the solution process that is presented in Section 2.1. Therefore, the solution of the T2FICCP model can be obtained, which is expressed as a set of intervals: $x_{jopt}^{+}=\left[x_{jopt}^{-},\;x_{jopt}^{+}\right]$ and $f_{opt}^{\pm }=\left[f_{opt}^{-},\;f_{opt}^{+}\right]$.

### 2.4. Solution Process

Based on the above analysis, the basic principles of solving the developed model are as follows: (1) the chance constrained constraint should be transformed into deterministic constraints with a feasible constraint set; (2) based on the interactive algorithm, the model, with T2FIS, could be transformed into two submodels; (3) each submodel can be transformed into its deterministic counterpart based on a type reduction technique; (4) The submodel corresponding to ${\tilde{f}}^{+}$ should be solved first because the objective is to be maximized; (5) The ${\tilde{f}}^{-}$ can be obtained based on the solution of the submodel corresponding to ${\tilde{f}}^{+}$.

The detailed solution process is explained as follows:

- Step 1: Establish the T2FICCP model.
- Step 2: Convert the chance constraint into the deterministic constraint with a feasible constraint set based on the CCP model.
- Step 3: Transform the model, which has converted the chance constraint into the deterministic constraint, into two submodels with T2FS based on the interactive algorithm.
- Step 4: Each submodel is converted into its deterministic counterpart by using a type reduction technique.
- Step 5: The submodel corresponding to ${\tilde{f}}^{+}$ is formulated and solved first based on the solution steps presented in Section 2.1.
- Step 6: Based on the solution of the first submodel, the submodel corresponding to ${\tilde{f}}^{-}$ is formulated and solved.
- Step 7: Based on the solutions of two submodels, the solution of the established T2FICCP model can be obtained, which is expressed as a set of intervals: $x_{jopt}^{+}=\left[x_{jopt}^{-},\;x_{jopt}^{+}\right]$ and $f_{opt}^{\pm }=\left[f_{opt}^{-},\;f_{opt}^{+}\right]$.
- Step 8: Repeat steps 3–7 corresponding to different $P_{i}$ levels.

## 3. Case Study

**Figure 1:**
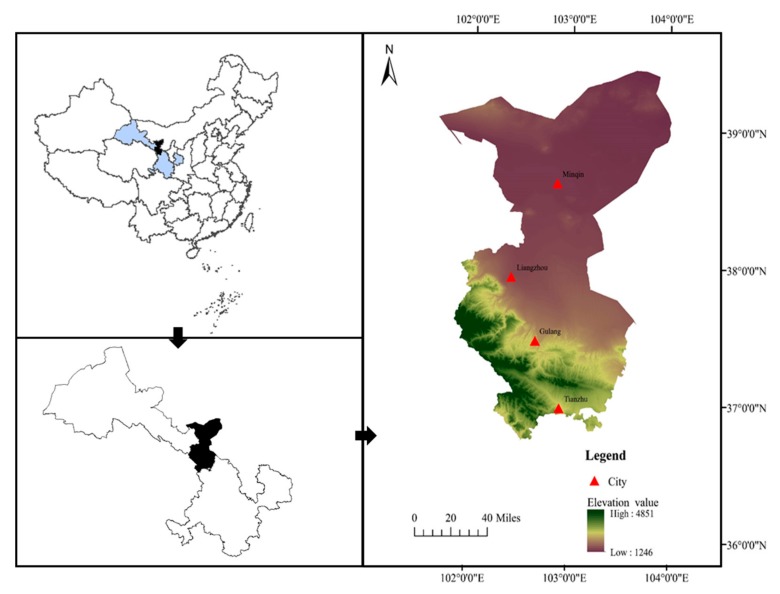
The study area.

The study area was selected is Wuwei City, located east of the Hexi Corridor in the central part of Gansu Province. It is the southern gate of the provincial capital, Lanzhou City (Figure 1) [2,36]. In addition, it is 326 km long from north to south and is 204 km wide (Figure 1) [28]. The land area (33,238 km^2^) that can be used for planting is very small, most of which is desert and mountainous land [37,40].

Wuwei City, is characterized by small per capita water resources and high per capita GDP, and it is located in the middle of the Shiyang River, which has a gradually decreasing amount of runoff [41]. As a result, it is one of the most extreme inland arid basins in China with a severely imbalanced water supply and demand and serious deterioration of the ecological environment. For example, the per capita water resources are 762 m^3^, which accounts for approximately 34.82% of the national level (2260 m^3^) [2].

However, the agricultural development of Wuwei depends heavily on water resources. As a result, a great amount of water resources are used for irrigation which accounts for approximately 88.28% of the total water consumption, even though the water shortage is becoming more and more serious [37,42]. Moreover, there is a strong relationship between the planting system and agricultural water consumption. In Wuwei’s planting system, the ratio of the grain crops to the economic crops is approximately 57.5:42.5 which means that the current planting industry is mainly involved with the grain crops [19].

Currently, the unreasonable planting structure badly restricts the development of the agricultural economy and has a great negative impact on the allocation of water resources when facing serious shortages of water resources. Therefore, it is significant to optimize the crop area and thus promote the optimal allocation of water resources for Wuwei City. Moreover, considering the multiple uncertainties and complex uncertain parameters in optimizing the allocation of crop areas, this paper established the T2FICCP model. Furthermore, the planting structure can be optimized based on the optimal schemes of the developed T2FICCP model.

In this study, wheat, maize, maize seed, bean, potato, cotton, oilseed, vegetable, cucurbit, apple and grape were chosen as the study crops.

### Model Building

**Figure 2:**
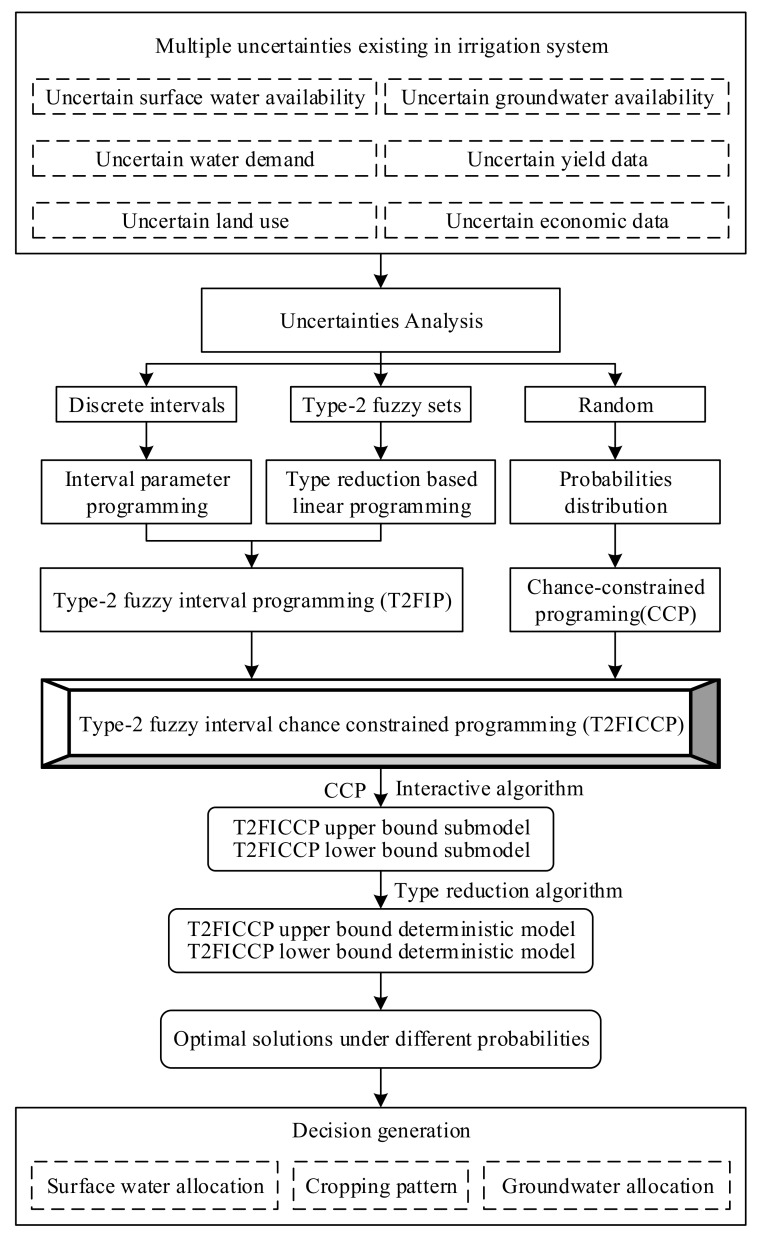
The research process of this study.

The conventional irrigation mode (broad irrigation) and water-saving mode both are used in the planting industry of Wuwei City. The former is widely adopted by local farmers, such as furrow irrigation. While the latter has a higher water use efficiency compared to the former mode, such as drip irrigation. To optimize the crop area effectively, different water saving modes were set in the study, leading to different water-saving scenarios. Based on the above analysis, an improved T2FICCP model was established for optimizing the allocation of the crop area considering multiple uncertainties and different water-saving scenarios. Figure 2 illustrates the research process of this study.

The formulation of the proposed model is presented below.

Objective functions:

Maximization of the economic benefit

(55) $$Max=\overset{11}{\underset{i=1}{\sum }}\left(P_{i1}^{\pm }\cdot Y_{i1}^{\pm }-C_{i1}\right)\cdot \left(1-\lambda_{i}\right)\cdot A_{i}^{\pm }+\overset{11}{\underset{i=1}{\sum }}\left(P_{i2}^{\pm }\cdot Y_{i2}^{\pm }-C_{i2}\right)\cdot \lambda_{i}\cdot A_{i}^{\pm }$$

Subject to:

Water resource availability constraint

(56) $$\overset{11}{\underset{1}{\sum }}\left(IW_{i1}^{\pm }\cdot A_{i}^{\pm }/IC\right)\cdot \left(1-\lambda_{i}\right)+\overset{11}{\underset{1}{\sum }}\left(IW_{i2}^{\pm }\cdot A_{i}^{\pm }/IC\right)\cdot \lambda_{i}+SW+TW+DW+EW≾\tilde{G}W^{\pm }+SFW^{P_{i}}$$

Water consumption constraint

(57) $$\overset{11}{\underset{i=1}{\sum }}ET_{i1}^{\pm }\cdot \left(1-\lambda_{i}\right)\cdot A_{i}^{\pm }+\overset{11}{\underset{i=1}{\sum }}ET_{i2}^{\pm }\cdot \lambda_{i}\cdot A_{i}^{\pm }\leq TET$$

Crop area constraint

(58) $$A_{i\;min}\leq A_{i}^{\pm }\leq A_{i\;max}$$

(59) $$\overset{11}{\underset{i=1}{\sum }}A_{i}^{\pm }\leq TA$$

Food security constraint

(60) $$FOD\cdot TPR\leq \overset{4}{\underset{i=1}{\sum }}A_{i}^{\pm }\cdot Y_{i1}^{\pm }\left(1-\lambda_{i}\right)+\overset{4}{\underset{i=1}{\sum }}A_{i}^{\pm }Y_{i2}^{\pm }\cdot \lambda_{i}$$

Nonnegativity constraint

(61) $$A_{i}^{\pm }\geq 0$$

where, *i* is the crop index (1 = wheat, 2 = maize, 3 = bean, 4 = potato, 5 = maize seed, 6 = cotton, 7 = oilseed, 8 = vegetable, 9 = cucurbit, 10 = apple, 11 = grape); 1 and 2 denote the conventional mode and water saving mode, respectively; $\lambda_{i}$ represents the ratio of the crop area under the water saving irrigation mode to the total area of crop *i*.

- $P_{i1}^{\pm }$: Price of crop *i* under the conventional irrigation mode (yuan/t) (Interval parameter);
- $P_{i2}^{\pm }$: Price of crop *i* under the water-saving irrigation mode (yuan/t) (Interval parameter);
- $Y_{i1}^{\pm }$: Yield of crop *i* under the conventional irrigation mode (t/ha) (Interval parameter);
- $Y_{i2}^{\pm }$: Yield of crop *i* under the water saving irrigation mode (t/ha) (Interval parameter);
- $C_{i1}$: The cost of crop *i* under the conventional irrigation mode (yuan/ha);
- $C_{i2}$: The cost of crop i under the water saving irrigation mode (yuan/ha);
- $A_{i}^{\pm }$: Irrigation area of crop *i* (10^4^ ha);
- $A_{i\;min}$: Minimized irrigation area of crop *i* (10^4^ ha);
- $A_{i\;max}$: Maximized irrigation area of crop *i* (10^4^ ha);
- *TA*: Total irrigation area of the study area (10^4^ ha);
- $IW_{i1}^{\pm }$: Irrigation quota of crop *i* under the conventional irrigation mode (m^3^/ha) (Interval number);
- $IW_{i2}^{\pm }$: Irrigation quota of crop *i* under the water saving irrigation mode (m^3^/ha) (Interval number);
- *IC*: Irrigation water use efficiency of the study area;
- *SW*: Water demand of the secondary industry (10^4^ m^3^);
- *TW*: Water demand of the tertiary industry (10^4^ m^3^);
- *DW*: Domestic water consumption (10^4^ m^3^);
- *EW*: Ecological water consumption (10^4^ m^3^);
- $\tilde{G}W^{\pm }$: Groundwater exploration (10^4^ m^3^) (Type-2 fuzzy interval parameter);
- *SFW*: Maximized surface water supply (10^4^ m^3^) (Random parameter);
- $ET_{i1}^{\pm }$: The ET of the *i*th crop under the conventional irrigation mode (m^3^/ha) (Interval parameter);
- $ET_{i2}^{\pm }$: The ET of the *i*th crop under the water saving irrigation mode (m^3^/ha) (Interval parameter);
- TET: The control indicator of the total water consumption (m^3^);
- *FDP*: Food demand per capita (t/p);
- *TPR*: Population of the study area (10^4^ p).

**Figure 3:**
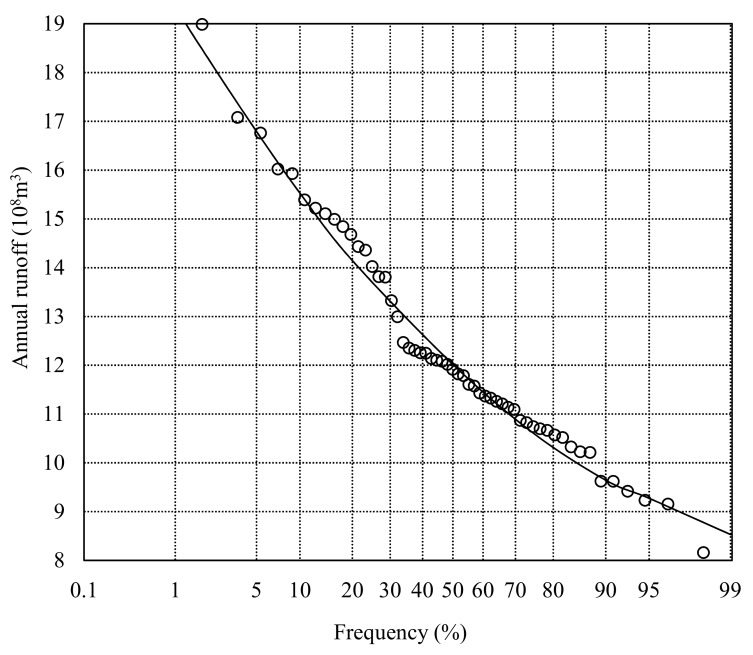
The P-III hydrographic curve.

In the established model, $\tilde{G}W^{\pm }$ has the characteristic of T2FI which can be solved by the T2IP method that is presented in the Section 2.1. In addition, in the water resources availability constraint, the maximized supply of the surface water has stochastic characteristics ($SFW^{P_{i}}$). In addition, the four violation probabilities, 9.23 × 10^8^ m^3^, 9.89 × 10^8^ m^3^, 10.36 × 10^8^ m^3^ and 10.64 × 10^8^ m^3^, were obtained based on the constructed P-III; hydrographic curve (Figure 3).

In addition, the corresponding data of the developed model was presented in Table 1 and Table 2.

## 4. Analysis of the Results and Discussion

**Figure 4:**
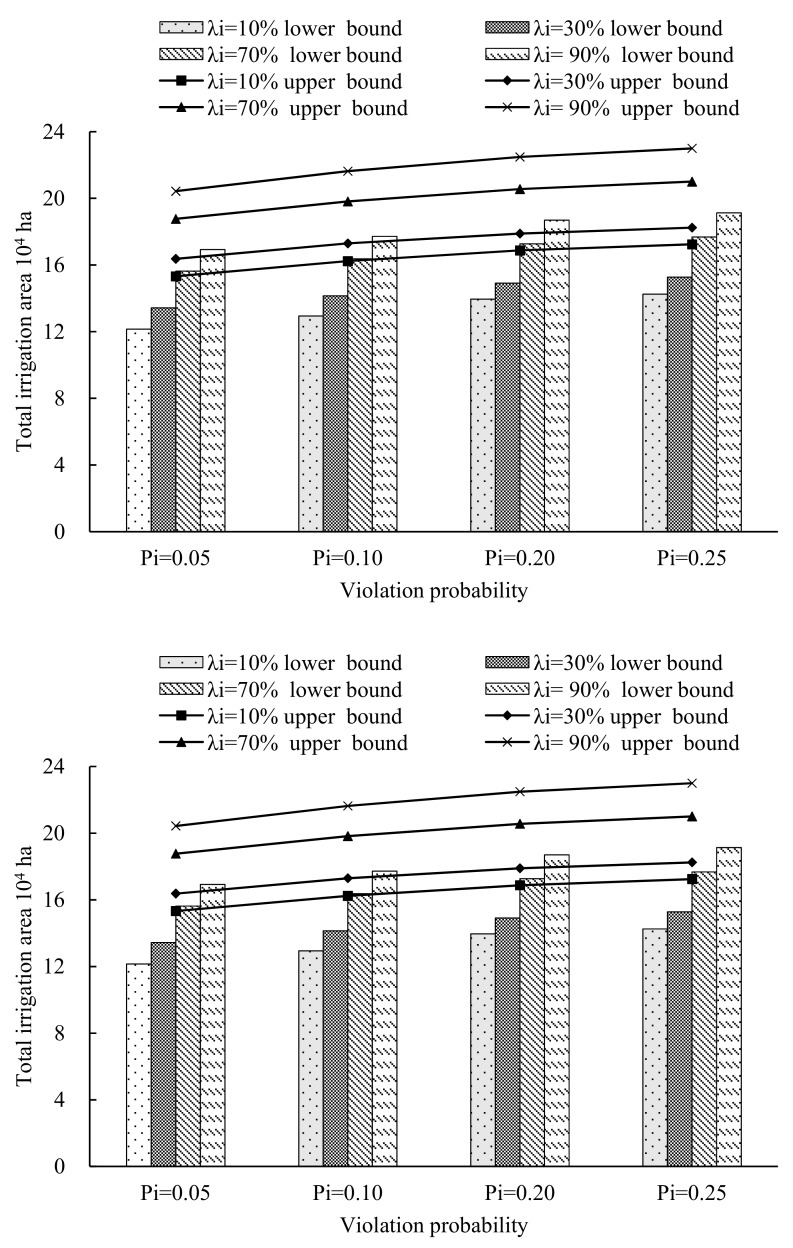
The total irrigation area of different water-saving modes under different violation probabilities, *P_i_*.

**Figure 5:**
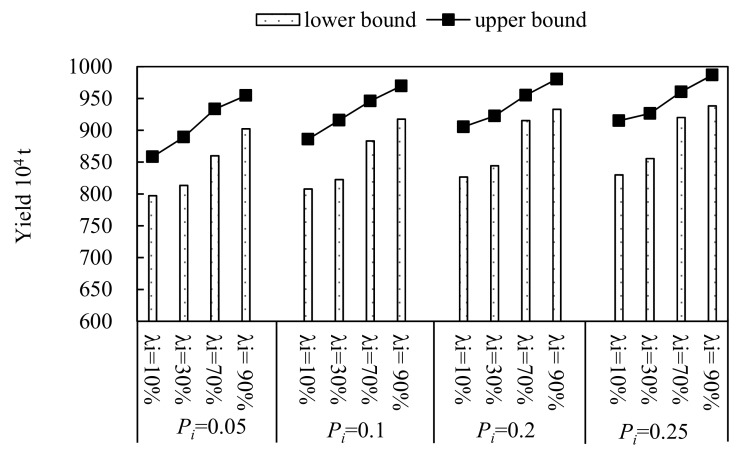
Yield of different water-saving modes under different violation probabilities, *P_i_*.

**Figure 6:**
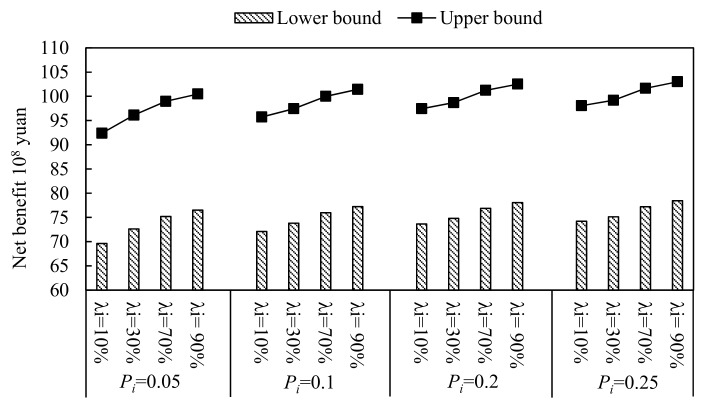
Net economic benefit of different water-saving modes under different violation probabilities, *P_i_*.

In this study, four water-saving scenarios with water-saving ratios of 10% 30%, 70% and 90%, were set (denoted as scenario 1 to 4, respectively). In addition, this study selected four violation probabilities, which were set as 0.05, 0.1, 0.2 and 0.25, and the corresponding available water resources were 0.23 × 10^8^ m^3^, 9.89 × 10^8^ m^3^, 10.36 × 10^8^ m^3^ and 10.64 × 10^8^ m^3^, respectively. Through solving the established model (10), the optimal crop area schemes were obtained under the different violation probabilities, *P_i_*, of each water-saving scenario. The results, which are generally expressed as intervals, can reflect more sensitivity. Figure 4, Figure 5 and Figure 6 present the optimized allocation of the crop area, yield and economic benefit under different violation probabilities of each water-saving scenario, respectively. From the figures, in general, the total irrigation area would vary under different water-saving scenarios and multiple uncertainties. For example, the lower bound of the total irrigation area would increase from 12.15 × 10^4^ ha (λ_i_ = 10%) to 17.30 × 10^4^ ha (λ_i_ = 90%) when the violation probability *P_i_* is 0.05. In addition, no matter the total irrigation area, yield or economic benefit, they have the same characteristic that the lower and upper bound increase as the water-saving levels and violation probabilities increase. When the result of the developed model is compared with the result of the model with conventional fuzzy uncertainty, there is a great difference. The biggest difference is that the lower bound and the upper bound of the result with the conventional fuzzy uncertainty have different trends, which represented that the upper bound value is decreasing while the lower bound value is increasing as the α-cut level increases. This is because of the triangular or trapezoidal membership function, which has the characteristic that the fuzzy extent weakens as the α-cut level increases, was usually selected as the membership function to deal with fuzzy uncertainty problems. However, in this study, in order to deal with the complex uncertainty expressed as type-2 fuzzy intervals, type-2 fuzzy interval programming was introduced into the established model. Therefore, based on the interactive algorithm and type reduction algorithm, the results are as shown in Figure 4, Figure 5 and Figure 6.

In addition, from Figure 4, Figure 5 and Figure 6, it can also be seen that that the total crop area, total yield and economic benefit would increase as water-saving level increases at the same violation probability. For example, the total crop area would increase from (12.94, 16.23) × 10^4^ ha (λ_i_ = 10%) to (17.89 × 10^4^, 21.63 × 10^4^) ×10^4^ ha (λ_i_ = 90%) when violation probability *P_i_* is 0.1. The result accords with the actual conditions. This is because the available water resources would increase as the water-saving levels increase. Thus, the crop area would increase as the available water resources increase, correspondingly. In addition, the total yield would increase from (807.69, 885.94) × 10^4^ t (λ_i_ = 10%) to (917.57, 969.72) × 10^4^ t (λ_i_ = 90%) when the violation probability, *P_i_*_,_ is 0.1. Furthermore, the economic benefit would increase from (69.61, 92.38) × 10^8^ ¥ (λ_i_ = 10%) to (76.51, 100.48) × 10^8^ ¥ (λ_i_ = 90%) when the violation probability *P_i_* is 0.1. This is because that there are positive relationships between the available water resources and the irrigation area, yield and economic benefit. As the water-saving levels increase, the available water resources would increase. Thus, the irrigation area, yield and economic benefit increase correspondingly.

Moreover, from the figures, it can be seen that the total crop area, total yield and economic benefit would vary as the violation probabilities varied, when the water-saving level was deterministic. For example, the total crop area would increase from (13.43, 16.37) ×10^4^ ha (*P_i_* = 0.05) to (15.28, 18.24) ×10^4^ ha (*P_i_* = 0.25) when the water-saving level is λ_i_ = 30%. Moreover, the total yield would increase from (813.50, 889.38) × 10^4^ t (*P_i_* = 0.05) to (855.58, 926.55) × 10^4^ t (*P_i_* = 0.25) when the water-saving level is λ_i_ = 30%. In addition, the economic benefit would increase from (72.63, 96.12) × 10^8^ ¥ (*P_i_* = 0.05) to (75.14, 99.19) × 10^8^ ¥ (*P_i_* = 0.25) when the water-saving level is λ_i_ = 30%.

From the above analysis, the crop area, yield and economic benefit would increase as the violation probabilities increased. This is because there is a positive relationship between the violation probabilities and available water resources, while there is negative relationship between the violation probabilities and the risk of water scarcity. Therefore, when the violation probabilities increase, the available water resources would increase, while the system stability would decrease. Therefore, the risk, which causes the water resource shortage, may be included in the chosen optimal schemes when achieving the given certain objective. Hence, the decision-makers should take the planning objective and water scarcity risk into consideration when they choose the desired schemes from a series of optimal plans under different violation probabilities. Thus, the knowledge of the decision maker and local situation would have a great influence on choosing the violation probabilities.

**Figure 7:**
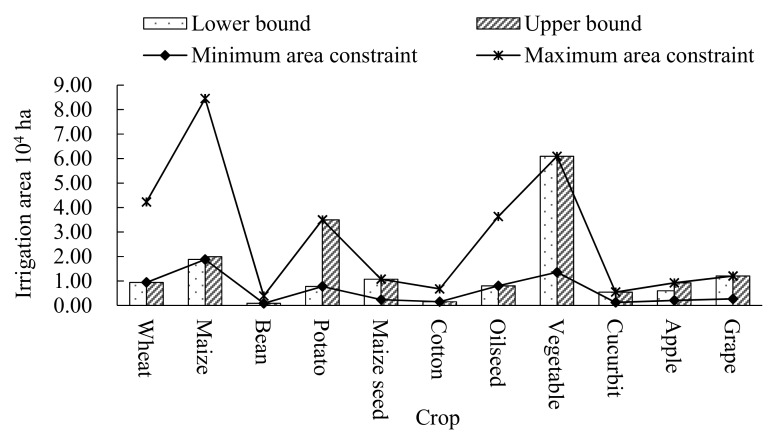
Optimal allocation area distribution for 11 crops (λi = 30%, *P_i_*= 0.1) and the maximum and minimum crop area constraints.

Furthermore, in order to represent the distribution of the irrigation areas of the 11 crops, the water-saving level of λ_i_ = 30% under *P_i_* = 0.1 was selected. From Figure 7, it shows that there are great differences in the irrigation areas of different crops. This is because some crops have a higher price or could produce a higher yield by using the same amount of water resources when compared with the other crops. For example, the yield per unit of maize under the conventional irrigation mode and water-saving mode are (10,470, 10,695) kg/ha and (12,750, 12,750) kg/ha, respectively, while the irrigation quota of maize under the conventional and water-saving mode are (5400, 5520) m^3^/ha and (3000, 3600) m^3^/ha, respectively. Furthermore, the price per unit and cost per unit under both irrigation modes are (1.3, 1.5) ¥/kg and 7788 ¥/ha, respectively. Therefore, when optimizing the irrigation water among the 11 crops, the minimum water resources constraint should be satisfied first. In addition, then the rest of the water resources would be assigned to the crops with a higher yield, higher prices, lower irrigation quota or lower cost.

**Figure 8:**
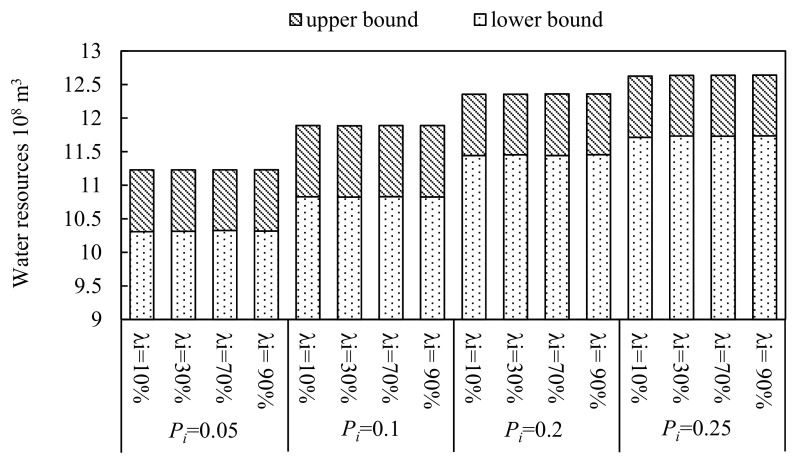
Irrigation water resources of different water-saving modes under different violation probabilities, *P_i._*

**Figure 9:**
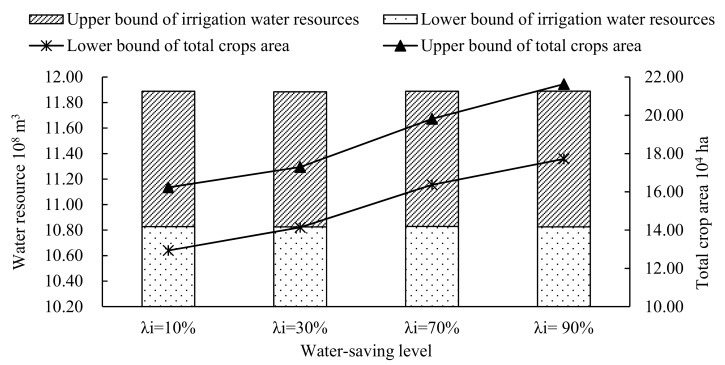
Irrigation water resource consumption and total crop area of different water-saving modes at violation probability *P_i_* = 0.1.

Figure 8 illustrates the distribution of the irrigation water resources under different violation probabilities in each water-saving scenario. As the figure shows, the total irrigation water consumption would not vary as the water-saving level increases under different violation probabilities, while as the violation probabilities increase, the total irrigation water consumption would increase. It means that the available water resources cannot satisfy the demand of the planting industry. In addition, Figure 9 represents the consumption of irrigation water resources and the total crop areas under different water-saving modes when the violation probability *P_i_* is 0.1. As the figure shows, as water-saving levels increases, the total area would increase, while the consumption of irrigation water resources would not vary. Figure 8 and Figure 9 show that the of water resource shortage is very serious and Wuwei’s water demand cannot be met, even if the violation probability reaches 0.25. Moreover, it also shows that the crop area, yield and economic benefit would increase as water-saving levels increases when the available water resources do not change. Therefore, it also suggests that attention should not only be paid to optimizing the allocation of crop areas but also focus on improving the water-saving levels which to make full use of the limited water resources.

Based on the above analysis, in this study, optimization management and water-saving scenarios were considered for improving the utilization efficiency of agricultural water resources. Moreover, in real-world problems, complex uncertainties and multiple uncertainties exist in the irrigation system. The developed T2FICCP model, incorporating type-2 fuzzy interval programming and chance-constrained programming, can effectively tackle the above problems. In addition, different water-saving scenarios were constructed in the study. Therefore, a range of optimal schemes for the irrigation water and irrigation area under different violation probabilities of different water-saving scenarios were given by the developed T2FICCP model. As different *Pi* values represent different levels of violation probability and different water-saving scenarios indicate different water-saving levels, the decision makers can choose sound decision schemes from all the optimal schemes.

## 5. Conclusions

In this paper, a T2FICCP model was developed to optimize the allocation of crop areas and irrigation water resources under multiple uncertainties and different water-saving scenarios. The developed model can effectively tackle uncertainties expressed as type-2 intervals and randomness by incorporating the type-2 fuzzy approach, interval parameter programming and chance-constrained programming within a general framework. In addition, as different water-saving scenarios were introduced, a range of optimization schemes under different water-saving levels of different violation probabilities could be represented by the proposed model.

The established model was then applied to a real-world case study in Wuwei City, Gansu Province, China. In this application, the maximum economic benefit was regarded as the objective, and the water resources constraint, ET constraint and food security constraint were considered. Furthermore, a series of crop area allocation results, which were under different violation probabilities of different water-saving scenarios, were generated and expressed as intervals. The analysis of the different crop area allocation policies corresponding to the various scenarios could provide decision makers with more choices according to specific conditions. However, one of the limitations of the developed T2FICCP model is that the multiobjective problem in the crop area planning system is not taken into account. Future work will extend the T2FICCP by introducing two-level programming, which will consider the requirements of the different water consumption sectors at different levels. In addition, the integration of the surface water, groundwater and other sources of water would be worthy of future research efforts to make the developed model more applicable in areas with multiple water sources.

## Figures and Tables

**Table 1:** The basic crop parameters.

Crops	Yield (kg/ha)		Irrigation Quota (m^3^/ha)		ET (m^3^/ha)		P (¥/ha)	C (¥/ha)	A_min_ (ha)	A_max_ (ha)
	Mode 1	Mode 2	Mode 1	Mode 2	Mode 1	Mode 2				
Wheat	(5447, 5745)	(5175, 5250)	(5150, 5250)	(4150, 4450)	(5410, 6490)	(4050, 4500)	(1.8, 2)	7179.5	0.9396	4.2282
Maize	(10,470, 10,695)	(12,750, 12,750)	(5400, 5520)	(3000, 3600)	(5400, 5520)	(3000, 3600)	(1.3, 1.5)	7788	1.8782	8.4519
Bean	(2482, 2884)	(2482, 2884)	(3950, 4450)	(3950, 4450)	(5715, 5715)	(5715, 5715)	4	5500	0.0866	0.3897
Potato	(27,000, 30,000)	33,000	(4370, 4850)	(3500, 3640)	(5200, 5705)	(4213, 4270)	(0.5, 0.6)	5250	0.7768	3.4956
Maize seed	(14,041, 14,428)	(13,835, 15,418)	(2250, 3250)	(2250, 2250)	(3621, 4248)	(3617, 3500)	2.2	16,075.5	0.2386	1.0737
Cotton	(1800, 1845)	(1980, 2030)	(3950, 4450)	(1950, 3150)	(3440, 4300)	(3600, 4695)	(5, 5.5)	5235	0.1500	0.6750
Oilseed	(3559, 3674)	(3559, 3674)	(4800, 5250)	(4800, 5250)	(3750, 4695)	(3750, 4695)	(3, 3.4)	3650	0.8018	3.6351
Vegetable	11,5500	11,3250	(4500, 5300)	(2680, 3100)	(5400, 6450)	(2200, 2900)	(1, 1.3)	18,000	1.3540	6.0930
Cucurbit	60,882	63,196	(4200, 4800)	(2700, 3150)	(3900, 4190)	(3300, 3670)	1.8	9125	0.1212	0.5454
Apple	11,545	11,545	(4780, 5290)	(4780, 5290)	(5825, 6742)	(5825, 6742)	(2, 2.2)	4950	0.2050	0.9225
Grape	(13,500, 15,000)	12,000	4130	2130	(4296, 5000)	(3017, 3073)	(3, 3.5)	8000	0.2670	1.2015

ET: evapotranspiration.

**Table 2:** The basic model parameters.

TA (10^4^ ha)	TPR (10^4^ P)	FDP (kg/P)	SW (10^4^ m^3^)	EW (10^4^ m^3^)	DW (10^4^ m^3^)	TW (10^4^ m^3^)	IC
24.28	186.14	300	21,838.5	15,544.8	5832.67	2261.57	0.59

TA: total irrigation area of study area; TPR: population of study area; FDP: food demand per capita; SW: Water demand of the secondary industry; EW: ecological water consumption; DW: domestic water consumption; TW: water demand of the tertiary industry; IC: irrigation water use efficiency of the study area.

## References

[1] Luo P., Zhou M., Deng H., Lyu J., Cao W., Takara K., Nover D., Schladow S.G. (2018). Impact of forest maintenance on water shortages: Hydrologic modeling and effects of climate change. Sci. Total Environ..

[2] Ren C.F., Li Z.H., Zhang H.B. (2019). Integrated multi-objective stochastic fuzzy programming and AHP method for agricultural water and land optimization allocation under multiple uncertainties. J. Clean. Prod..

[3] Wang S., Huang G.H. (2015). A multi-level Taguchi-factorial two-stage stochastic programming approach for characterization of parameter uncertainties and their interactions: An application to water resources to water resources management. Eur. J. Oper. Res..

[4] Li M., Fu Q., Singh V.P., Liu D. (2018). An interval multi-objective programming model for irrigation water allocation under uncertainty. Agric. Water Manag..

[5] Luo P.P., He B., Duan W.L. (2018). Impact assessment of rainfall scenarios and land-use change on hydrologic response using synthetic area IDF curves. J. Flood Risk Manag..

[6] Cai X.L., Cui Y.L. (2009). Crop Planting structure extraction in irrigated areas from multi-sensor and multi-temporal remote sensing data. Trans. Chin. Soc. Agric. Eng..

[7] Singh A. (2015). Land and water management planning for increasing farm income in irrigated dry areas. Land Use Policy.

[8] Mohammed M., Ashim D.G., Pushpa R.O. (1997). Optimal crop planning model for an existing groudwater irrigation project in Thailand. Agric. Water Manag..

[9] Sarker R.A., Quaddus M.A. (2002). Modelling a nationwide crop planning problem using a multiple criteria decision making tool. Comput. Ind. Eng..

[10] Belin B., Kodal S. (2003). A non-linear model for farm optimization with adequate and limited water supplies application to the south-east Anatolian project (GAP) region. Agric. Water Manag..

[11] Gorantiwar S.D., Smout I.D. (2005). Multilevel approach for optimizing land and water resources and irrigation deliveries for tertiary units in large irrigation schemes. II: Application. J. Irrig. Drain. Eng..

[12] Singh A. (2014). Optimizing the use of land and water resources for maximizing farm income by mitigating the hydrological imbalance. J. Hydrol. Eng..

[13] Garg N.K., Dadhich S.M. (2014). Integrated non-linear model for optimal cropping pattern and irrigation scheduling under deficit irrigation. Agric. Water Manag..

[14] Chang J., Guo A., Wang Y., Ha Y., Zhang R., Xue L., Tu Z. (2019). Reservoir operations to mitigate drought effects with a hedging policy triggered by the drought prevention limiting water level. Water Resour. Res..

[15] Nguyen D.C., Dandy G.C., Maier H.R., Ascough J.C. (2016). Improved ant colony optimization for optimal crop and irrigation water allocation by incorporating domain knowledge. J. Water Resour. Plan. Manag..

[16] Galan-Martin A., Vaskan P., Anton A., Esteller L.J., Guillén-Gosálbez G. (2017). Multi-objective optimization of rainfed and irrigated agricultural areas considering production and environmental criteria: A case study of wheat production in Spain. J. Clean. Prod..

[17] Fazlali A., Shourian M. (2018). A demand management based crop and irrigation planning using the simulation-optimization approach. Water Resour. Manag..

[18] Li M., Fu Q., Singh V.P., Ji Y., Liu D., Zhang C., Li T. (2019). An optimal modelling approach for managing agricultural water-energy-food nexus under uncertainty. Sci. Total Environ..

[19] Li M., Fu Q., Singh V.P., Ma M.W., Liu X. (2017). An intuitionistic fuzzy multi-objective non-linear programming model for sustainable irrigation water allocation under the combination of dry and wet conditions. J. Hydrol..

[20] Hamid R.S., Mohammad A.A. (2011). Optimal crop planning and conjunctive use of surface water and groundwater resources using fuzzy dynamic programming. J. Irrig. Drain. Eng..

[21] Tong F.F., Guo P. (2013). Simulation and optimization for crop water allocation based on crop water production functions and climate factor under uncertainty. Appl. Math. Model..

[22] Xie Y.L., Huang G.H., Li W., Li J.B., Li Y.F. (2013). An inexact two-stage stochastic programming model for water resources management in Nansihu Lake Basin, China. J. Environ. Manag..

[23] Wang S., Huang G.H. (2014). An integrated approach for water resources decision making under interactive and compound uncertainties. Omega.

[24] Jiang Y., Xu X., Huang Q., Huo Z., Huang G. (2016). Optimizing regional irrigation water use by integrating a two-level optimization model and an agro-hydrological model. Agric. Water Manag..

[25] Li Y.P., Huang G.H. (2009). Fuzzy-stochastic-based analysis method for planning water resources management systems with uncertain information. Inf. Sci..

[26] Li Y.P., Liu J., Huang G.H. (2014). A hybrid fuzzy-stochastic programming method for water trading within an agricultural system. Agric. Syst..

[27] Wang C.X., Li Y.P., Huang G.H., Zhang J.L. (2016). A type-2 fuzzy interval programming approach for conjunctive use of surface water and groundwater under uncertainty. Inf. Sci..

[28] Ren C.F., Zhang H.B. (2018). A fuzzy max-min decision bi-level fuzzy programming model for water resources optimization allocation under uncertainty. Water.

[29] Aliev R.A., Pedrycz W., Kreinovich V., Huseynov O.H. (2016). The general theory of decisions. Inf. Sci..

[30] Kundu P., Kar S., Maiti M. (2014). Fixed charge transportation problem with type-2 fuzzy variables. Inf. Sci..

[31] Nie S., Huang C.Z., Huang G.H., Li Y.P., Chen J.P., Fan Y.R., Cheng G.H. (2016). Planning renewable energy in electric power system for sustainable development under uncertainty—A case study of Beijing. Appl. Energy.

[32] Li Y.P., Huang G.H., Yang Z.F., Nie S.L. (2008). IFMP: Interval-fuzzy multistage programming for water resources management under uncertainty. Resour. Conserv. Recycl..

[33] Xie Y.L., Xia D.X., Ji L., Huang G.H. (2018). An inexact stochastic-fuzzy optimization model for agricultural water allocation and land resources utilization management under considering effective rainfall. Ecol. Indic..

[34] Zhu H., Huang G.H. (2011). SLFP: A stochastic linear fractional programming approach for sustainable waste management. Waste Manag..

[35] Ren C.F., Guo P., Li M., Gu J.J. (2013). Optimization of industrial structure considering the uncertainty of water resources. Water Resour. Manag..

[36] Ren C.F., Li R.H., Zhang L.D., Guo P. (2016). Multiobjective stochastic fractional goal programming model for water resources optimal allocation among industries. J. Water Resour. Plan. Manag..

[37] Ren C.F., Guo P., Tian Q., Zhang L.D. (2017). A multi-objective fuzzy programming model for optimal use of irrigation water and land resources under uncertainty in Gansu Province, China. J. Clean. Prod..

[38] Zara Y., Daneshmand A. (1995). A linear approximation method for solving a special class of the chance constrained programming problem. Eur. J. Oper. Res..

[39] Charnes A., Cooper W.W., Kriby P. (1972). Chance constrained programming an extension of statistical method. Optimizing Methods in Statistics.

[40] Gui Z.Y., Li M., Guo P. (2016). Simulation-based inexact fuzzy semi-infinite programming method for agricultural cultivated area planning in the Shiyang River Basin. J. Irrig. Drain. Eng..

[41] Xu W.H., Zhong Y.M., Chen G. (2007). The state of the art in water resources utilization in Shiyang River Basin and the countermeasures for sustainable utilization. J. Glaciol. Geocryol..

[42] Huang Y., Li Y.P., Chen X., Ma Y.G. (2012). Optimization of the irrigation water resources for agricultural sustainability in Tarim river basin, China. Agric. Water Manag..

